# Predictive Values of Preoperative Tests for Difficult Laryngoscopy and Intubation in Adult Patients at Tikur Anbessa Specialized Hospital

**DOI:** 10.1155/2019/1790413

**Published:** 2019-04-01

**Authors:** Tadese Tamire, Habtamu Demelash, Wosenyeleh Admasu

**Affiliations:** ^1^College of Health Sciences, Department of Anaesthesia, Debre Tabor University, Debra Tabor, Ethiopia; ^2^College of Health Sciences, School of Medicine, Department of Anaesthesia, Addis Ababa University, Addis Ababa, Ethiopia

## Abstract

**Introduction:**

The significance of difficult or failed tracheal intubation following induction is a well-recognized cause of morbidity and mortality in anesthetic practice. Nevertheless, the need to predict potentially difficult tracheal intubation has received a little attention. During routine anesthesia, the incidence of difficult tracheal intubation has been estimated at 1.5%–8% of general anesthetics. Difficulties in intubation have been associated with serious complications, such as brain damage or death, particularly when failed intubation has occurred. Occasionally, in a patient with a difficult airway, the anesthetist is faced with the situation where mask ventilation proves difficult or impossible. This is one of the most critical emergencies that may be faced in the practice of anesthesia. If the anesthetist can predict which patients are likely to prove difficult to intubate, he/she may reduce the risks of anesthesia considerably. In Ethiopia, there are no data on the magnitude of difficult laryngoscopic tracheal intubation and no standard guidelines for preoperative tests. The main concern of this study was to provide information on the magnitude of difficult laryngoscopic intubation and to determine valuable preoperative tests to predict difficult laryngoscopy and intubation in patients with apparently normal airways which can help anesthetists to improve preoperative airway assessment and contribute to decrease anesthesia-related morbidity and mortality.

**Objective:**

The main objective of this study was to assess the magnitude and predictive values of preoperative tests for difficult laryngoscopy and intubation, among surgical patients who underwent elective surgery under general anesthesia with endotracheal intubation in Tikur Anbessa Hospital from February 1 to March 30, 2016.

**Study Design:**

A facility-based cross-sectional study design was used.

**Result:**

In this study, we found the magnitude of difficult laryngoscopy and intubation as 13.6% and 5%, respectively. 33.3% of patients with difficult laryngoscopy were found to be difficult for intubation. Mallampati test, interincisor distance, and thyromental distance were identified to be good preoperative tests to predict difficult laryngoscopic intubation when used in combination.

**Recommendation:**

We recommend anesthesia professionals to use combination of MMC/TMD/IID for their routine preoperative airway assessment.

## 1. Introduction

### 1.1. Background Information

The term “airway” in its day-to-day usage refers to the upper airway which may be defined as the extrapulmonary air passage, consisting of the nasal and oral cavities, pharynx, larynx, trachea, and large bronchi. “Difficult airway” is one in which there is a problem in establishing or maintaining gas exchange via a mask, an artificial airway, or both. Recognizing the potential for a difficult airway (DA) in designated “Difficult airway clinics” allows time for optimal preparation, proper selection of equipment and technique, and participation of personnel experienced in difficult airway management [1, 2]. For securing the airway, tracheal intubation using direct laryngoscopy remains the method of choice in most cases. However, difficult laryngoscopic tracheal intubation occurs in 1.5%–8% of general anesthetics [3]. Difficulty in airway management is a major cause of morbidity and mortality in anesthetic practice [4].

Difficult laryngoscopy (poor glottis visualization) is considered as a surrogate indicator of difficult intubation [2]. The ability to identify patients at risk of difficult tracheal intubation is important especially in patients with apparently normal airways. The diagnostic accuracy of the screening tests varies between different studies. This is attributed to the difference in the incidence of difficult laryngoscopy and intubation, inadequate statistical power, different test thresholds, and differences in patient characteristics. Differences in patient characteristics may also influence the incidence of difficult laryngoscopy and difficult intubation [4].

The anesthesia preoperative evaluation is the clinical foundation and framework of perioperative patient management and can potentially reduce operative morbidity and enhance patient outcomes [5]. Parameters, such as interincisor distance (IID), mandibular protrusion (MP), thyromental distance (TMD), sternomental distance (SMD), oropharyngeal space (modified Mallampati class), and grade of laryngoscopic view, are the most commonly used preoperative tests that can assist to predict difficult intubation. For each of these parameters, there are several airway measures available, and their reliability and predictive ability varies widely. This study will assess the magnitude and validity of airway parameters in predicting difficult laryngoscopic intubation for general anesthesia (GA) in adults and effect of combining the parameters on the validity.

### 1.2. Statement of the Problem

Black lion hospital (Tikur Anbessa in Amharic), located in the nation's capital Addis Ababa, is Ethiopia's largest public hospital. In 1998, TASH which is also the largest referral hospital in the country was given to Addis Ababa University by the Ministry of Health (MOH) for the health faculty as a main teaching hospital. The faculty is the oldest and the largest among the health training institutions in the country, staffed with the most senior specialists. The hospital provides a tertiary level referral treatment. It offers diagnosis and treatment for approximately 370,000–400,000 patients a year. The hospital has 800 beds and 17 operation rooms. But due to reformation, currently only 8 operation rooms are functional, and approximately 7000–9000 patients undergo surgery in a year including cesarean section and emergency surgery.

Expertise in airway management is essential in every medical specialty. Maintaining a patent airway is essential for adequate oxygenation and ventilation, and failure to do so, even for a brief period of time, can be life threatening. Respiratory events are the most common anesthetic related injuries. The three main causes of respiratory-related injuries are inadequate ventilation, esophageal intubation, and difficult tracheal intubation. Difficult tracheal intubation accounts for 17% of the respiratory-related injuries and results in significant morbidity and mortality. In fact, up to 28% of all anesthesia-related deaths are secondary to the inability to mask ventilation or intubation [2]. The incidence of difficult laryngoscopy and intubation in Indian patients was 9.7% and 4.5%, respectively [4].

Unanticipated difficult laryngoscopic intubation places patients at increased risk of complications ranging from sore throat to serious airway trauma, brain damage, and even death. These complications are probably the result of a lack of accurate predictive tests for difficult intubation and inadequate preoperative examinations of the airway [4].

Because difficult or failed endotracheal intubations are one of the leading causes of anesthesia-related morbidity and mortality in anesthetized patients, there is a need for accurate tests to predict difficult intubation [6, 7]. The ability to identify patients at risk of difficult tracheal intubation is important especially in patients with apparently normal airways. There are no standard guidelines for preoperative tests in most of Ethiopian hospitals.

Preoperative assessment of various anatomic and clinical features helps in identifying potentially difficult laryngoscopy [4]. However, the diagnostic accuracy of screening parameters varies between different studies because of patients' ethnic group, sex, and physical and medical characteristics. For example, in Asian patients, it may be more difficult to intubate the trachea than in Caucasians [3, 4]. Moreover, most studies do not provide a “measure” of difficult intubation in patients with difficult laryngoscopy. This is attributed to the difference in incidence of difficult laryngoscopy and intubation and inadequate statistical power of different tests. In addition to this, none of the studies of difficult laryngoscopy and intubation have been performed in Ethiopia.

This study can provide information on the magnitude of difficult laryngoscopy and intubation and predictive value of common preoperative tests which will contribute in the improvement of preoperative airway assessment to decrease anesthesia-related morbidity and mortality.

### 1.3. Literature Review

Difficult laryngoscopy is inability to view the glottis opening using a conventional curve blade laryngoscope, corresponding to a Cormack and Lehane III or IV grade view, in which only epiglottis or only pharynx and tongue, respectively, may be visualized, whereas difficult intubation is defined as usage of direct laryngoscopy taking more than 3 attempts or more than 10 minutes to complete tracheal intubation by the trained anesthetist [1, 2, 6]. Difficult or failed endotracheal intubation is one of the leading causes of anesthesia-related morbidity and mortality [1, 2, 4, 6].

The unanticipated difficult airway occurs with a low but consistent incidence in anesthesia practice. Literature review from 1990 to 1996 reported as difficult direct laryngoscopy occurs in 1.5–8.5% of general anesthetics, and difficult intubation occurs with a similar incidence [3, 8].

Keyvan et al. [6] conducted an observational study at the tertiary-care level hospital to predict difficult intubation. Of the 461 patients included in this analysis, 38/461 (8.24%) were classified as difficult to intubate [6]. Another recent prospective study conducted by Smita et al. [4] in India on 330 adult patients to assess the incidence of difficult laryngoscopy and intubation in the Indian population reported the incidence of difficult laryngoscopy and intubation as 9.7% and 4.5%, respectively [4]. A more recent study by Bhavdip et al. showed the incidence of difficult endotracheal intubation as 8.1% [7].

Because failed endotracheal intubation is a principal cause of morbidity and mortality in anesthetized patients, there is a need for accurate tests to predict difficult intubation [9]. This risk can be reduced if patient's airway is evaluated preoperatively [7].

Mostly cited preoperative tests to predict the absence or presence of difficult laryngoscopy and intubation are as follows: modified Mallampati class (MMC), interincisor distance (IID), thyromental distance (TMD), sternomental distance (SMD), mandibular protrusion, and Cormack and Lehane laryngoscopic grade [4, 5, 7–9]. These tests are very cheap, simple, and easily applicable in clinical practice by any level of anesthesia professionals.

Assessment techniques which utilize multiple characteristics to derive a risk factor tend to be more accurate predictors. Literatures have recommended that the use of combined preoperative measurement parameters in predicting difficult intubation. However, limited information is available on effect of combining these parameters in enhancing the validity of parameters.

Savva [10] conducted a prospective study to predict difficult tracheal intubation on 350 patients, and he found difficult tracheal intubation in 17/350 (4.9%) patients, of whom four (1.14%) had a grade III or IV view on laryngoscopy. A sternomental distance of 12.5 cm or less with the head fully extended on the neck and the mouth closed predicted 14 of the 17 patients in whom tracheal intubation was difficult. As a screening test, sternomental distance appeared to be more sensitive (82.4%) and more specific (88.6%) than thyromental distance (64.7% and 81.4%, respectively), the modified Mallampati test (64.7% and 66.1%, respectively), and forward protrusion of the mandible (29.4% and 85.0%, respectively) [10].

Tse et al. [9] conducted a prospective blind study to determine whether a difficult endotracheal intubation could be predicted preoperatively by evaluation of one or more anatomic features of the head in St. Elizabeth's Medical Center of Boston. A total of 471 patients (220 men and 251 women aged 18–89 year) were enrolled in the study. Sixty-two of them were found at laryngoscopy to have airways that were difficult to intubate (laryngoscopy grade III or IV). There were no failed intubations. Assignment to oropharyngeal class 3, a thyromental distance ≤7 cm, and a head extension ≤80 degrees were selected as indicators of difficult intubation [9]. Keyvan et al. [6] conducted observational study at the tertiary-care level hospital to predict difficult intubation. Of the 461 patients included in this analysis, 38 were classified as difficult to intubate. Multivariable analysis identified three airway tests that were highly significant for predicting difficult tracheal intubation. These were as follows: (1) “mouth opening,” (2) “chin protrusion,” and (3) “atlanto-occipital extension.” Using these tests, a validated, highly reliable, and predictive model is produced to determine the probability of difficult intubation for patients.

Patients requiring endotracheal intubation in the resuscitation room of a UK teaching hospital between June 2002 and September 2003 were assessed on criteria based on the LEMON method. At laryngoscopy, the Cormack and Lehane grade was recorded. An airway assessment score was devised and assessed. 156 patients were intubated during the study period. There were 114 Cormack and Lehane grade 1 intubation, 29 grade 2 intubations, 11 grade 3 intubations, and 2 grade 4 intubations. Patients with large incisors (*p*=0.001), a reduced interincisor distance (*p*=0.05), or a reduced thyroid to floor of mouth distance (*p*=0.05) were all more likely to have a poor laryngoscopic view (grades 2, 3, or 4). Patients with a high airway assessment score were more likely to have a poor laryngoscopic view compared with those patients with a low airway assessment score (*p*=0.05). An airway assessment score based on criteria of the LEMON method is able to successfully stratify the risk of intubation difficulty in the emergency department. Patients with a poor laryngoscopic view (grades 2, 3, or 4) were more likely to have large incisors, a reduced interincisor distance, and a reduced thyroid to floor of mouth distance [11].

Iohom et al. [12] conducted a study on a total of 212 (109 male, 103 female) nonobstetric surgical patients, aged >18 yr, undergoing elective surgical procedures requiring tracheal intubation by assessing preoperatively with respect to the oropharyngeal (modified Mallampati) classification and thyromental and sternomental distances. An experienced anesthetist, blinded to the preoperative airway assessment, performed laryngoscopy and graded the view according to Cormack and Lehane's classification. Twenty tracheal intubations (9%) were difficult, as defined by a Cormack and Lehane grade 3 or 4, or the requirement for a bougie in patients with Cormack and Lehane grade 2. Used alone, the Mallampati oropharyngeal view and thyromental and sternomental distances were associated with poor sensitivity, specificity, and positive predictive values. Combining the Mallampati class III or IV with either a thyromental distance <6.5 cm or a sternomental distance <12.5 cm decreased the sensitivity (from 40 to 25 and 20%, respectively) but maintained a negative predictive value of 93%. The specificity and positive predictive values increased from 89 to 27%, respectively, for Mallampati alone to 100%. The findings suggest that the Mallampati classification, in conjunction with measurement of the thyromental and sternomental distances, may be a useful routine screening test for preoperative prediction of difficult tracheal intubation [12].

Merah et al. [13] found difficulty to visualize the larynx in 13/380 (3.4%) patients. The sensitivity, specificity, and the positive predictive value for the five airway predictors were as follows: MMT (61.5%; 98.4%; 57.1%), TMD (15.4%; 98.1%; 22.2%), SMD (0%; 100%; 0%), HLM (30.8%, 76.0%; 4.3%), and IIG (30.8%; 97.3%; 28.6%). The best combination of predictors was MMT/TMD/IIG with a sensitivity, specificity, and positive predictive value of 84.6%, 94.6%, and 35.5%, respectively. Logistic regression analysis showed that weight, MMT, IIG, and TMD was independent predictors of DVL. They concluded that MMT, TMD, and IIG appear to provide the optimal combination in prediction of DVL in a West African population [13].

On the contrary, Khan et al. [14] conducted a prospective study and showed the prevalence of difficult intubation as 5% (*n*=19). Class III ULBT, IID < 4.5 cm, TMD < 6.5 cm, and SMD < 13 cm were defined as predictors of difficult intubation. The specificity and accuracy of the ULBT were significantly higher than TMD, SMD, and IID individually (specificity was 91.69%, 82.27%, 70.64%, and 82.27%, respectively, and accuracy was 91.05%, 71.32%, 81.84%, and 76.58%, respectively). The combination of the ULBT with SMD provided the highest sensitivity. They concluded that the specificity and accuracy of the ULBT are significantly higher than the other tests and are more accurate in airway assessment. However, the ULBT in conjunction with the other tests could more reliably predict easy laryngoscopy or intubation [14].

Another prospective study conducted by Smita et al. [4] in India on 330 adult patients to assess the incidence of difficult laryngoscopy and intubation in the Indian population showed the incidence of difficult laryngoscopy and intubation as 9.7% and 4.5%, respectively. Univariate analysis showed that increasing age and weight, male gender, modified Mallampati class (MMC) 3 and 4 in sitting and supine positions, interincisor distance (IID) ≤3.5 cm, thyromental (TMD) and sternomental distance, ratio of height and TMD, short neck, limited mandibular protrusion, decreased range of neck movement, history of snoring, receding mandible, and cervical spondylosis were associated with difficult laryngoscopy. Multivariate analysis identified four variables that were independently associated with difficult laryngoscopy: MMC class 3 and 4, range of neck movement <80°, IID ≤ 3.5 cm, and snoring [4].

A more recent prospective study by Bhavdip et al. [7] conducted to assess the validity of different parameters in predicting difficult intubation for general anesthesia (GA) in adults and effect of combining the parameters on the validity on 135 patients showed that the sensitivity and specificity of MMT as 28.6% and 93%, respectively. The TMD (<6.5 cm) had sensitivity and specificity of 100% and 75.8%, respectively. The SMD (<12.5 cm) had sensitivity and specificity of 91% and 92.7%, respectively. Combination of MMT grading and TMD and SMD measurements increased the validity (sensitivity of 100% and specificity of 92.7%). They found high specificity in MMT but suggested that the validity of using combination of MMT, SMD, and TMD as compared to MMT alone is very high in predicting difficult intubation in adult patients [7].

As we can see from the above literatures, the diagnostic accuracy of screening tests varies between different studies, and most of the researchers recommended the combination of different preoperative tests to increase the validity of parameters in predicting difficult laryngoscopy and intubation. However, in Tikur Anbessa specialized Hospital, there are no guidelines for preoperative airway assessment and no study is conducted to assess the magnitude and predictive value of preoperative tests even at the national level. There is also limited data on the predictive value of difficult laryngoscopy for difficult intubation.

The incidence of difficult laryngoscopy and intubation is not the same in different studies; this can be due to patients' ethnic group, physical and medical characteristics, skill of the anesthetists, and the diagnostic tests used. For example, in Asian patients, it may be more difficult to intubate the trachea than in Caucasians [9, 15].

**Figure 1:**
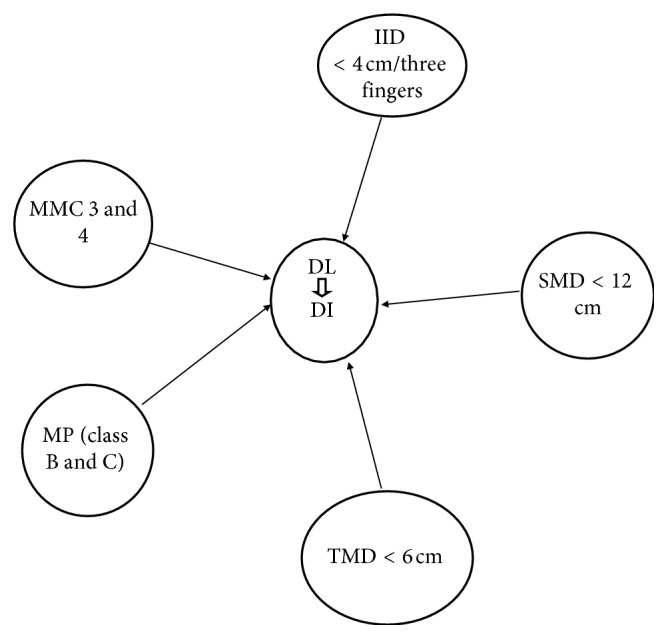
Conceptual framework.

This prospective observational study was used to assess the magnitude of difficult laryngoscopy and intubation and predictive value of preoperative tests in estimating difficult laryngoscopic intubation among elective surgical patients in Tikur Anbessa Specialized Hospital from February 1 to March 30, 2016. The study also tested the effect of combining different parameters on their validity in predicting difficult intubation. This study is expected to provide information that can help to develop guidelines for preoperative evaluation of patients and can contribute for the improvement of quality of anesthesia care. This study can also be used as baseline data for researchers to carry out a multicenter study at a national level (Figure 1).

## 2. Significance of the Study

Even though difficulty in airway management is a major cause of morbidity and mortality in anesthetic practice, attention is not given for prevalence of this problem and improvement of preoperative airway assessment. The diagnostic accuracy of preoperative tests varies between different studies. This is attributed to the difference in the incidence of difficult laryngoscopy and intubation among different populations. Differences in the patient characteristics, clinical setup, and skill of the anesthetists can also influence the magnitude of difficult laryngoscopy and intubation. In addition to this, there are no published data on the magnitude or prevalence and predictive values of preoperative tests in our country, even at a national level. Moreover, most studies do not provide a “measure” of difficult intubation in patients with difficult laryngoscopy.

Evidence-based local or national data on the magnitude of the problem and normal values of preoperative tests will help anesthetists to improve quality of anesthesia care. The purpose of this study is therefore to provide evidence-based information to the anesthetists and other concerned professionals on the magnitude of difficult laryngoscopy and intubation and to describe the validity of clinically useful preoperative tests for predicting difficult laryngoscopy and difficult intubation in patients with seemingly normal airway in Tikur Anbessa Specialized Hospital from February 1 to March 30, 2016. This study will contribute to the improvement of anesthesia quality care by decreasing morbidity and mortality of patients associated with endotracheal intubation. It can also be used as baseline data for further multicenter studies and for the development of guidelines for preoperative airway assessment to predict difficult laryngoscopic tracheal intubation.

## 3. Objectives

### 3.1. General Objective

To assess the magnitude and predictive values of preoperative tests for difficult laryngoscopy and intubation among surgical patients who underwent elective surgery under general anesthesia in Tikur Anbessa Specialized Hospital from February 1 to March 30, 2016, Addis Ababa, Ethiopia.

### 3.2. Specific Objectives

1. To assess the magnitude of difficult laryngoscopy and intubation among surgical patients who underwent elective surgery under general anesthesia in Tikur Anbessa Specialized Hospital
2. To determine the predictive values of preoperative tests for difficult laryngoscopy and intubation among surgical patients who underwent elective surgery under general anesthesia in Tikur Anbessa Specialized Hospital
3. To describe the effect of combining preoperative tests on the validity of predicting difficult laryngoscopy and intubation among surgical patients who underwent elective surgery under general anesthesia in Tikur Anbessa Specialized Hospital

## 4. Methods

### 4.1. Study Design

A facility-based cross-sectional study design was used.

### 4.2. Study Area and Period

This study was conducted in Addis Ababa University, college of health sciences, Tikur Anbessa Specialized Teaching Hospital. This hospital is found in the heart of Addis Ababa and is the largest teaching hospital in Ethiopia, having about 800 beds and 17 operation rooms. It provides diagnosis and treatment for 370,000–400,000 patients per year and approximately 7000–9000 patients undergo surgery in a year. The study was conducted from February 1 to March 30, 2016.

### 4.3. Study Population

#### 4.3.1. Source Population

All surgical patients who underwent elective surgery under general anesthesia were selected.

#### 4.3.2. Study Population

All surgical patients who were scheduled for elective surgery under general anesthesia with endotracheal intubation were included.

### 4.4. Eligibility Criteria

#### 4.4.1. Inclusion Criteria

All elective surgical patients scheduled for surgery under GA with endotracheal intubation were included.

#### 4.4.2. Exclusion Criteria

The following patients were excluded from this study:

- Patients with known airway difficulty due to trauma and congenital or acquired abnormalities that compromise the airway.
- Patients with goiter because they are already at increased risk of difficult airway.
- Obstetrics due to anatomic and physiological changes of airway during pregnancy predispose them to difficult airway, and the use of rapid sequence induction with cricoid pressure may lead to difficult intubation. So, this may affect the validity of preoperative tests.
- Critically ill patients in which airway assessment is difficult.
- Age less than 18 years because of the anatomic differences of the adult and pediatric airway.
- Psychiatric patients because they may not cooperate for the airway assessment.

### 4.5. Sample Size Determination

Sample size was determined using the finite population correction formula by assuming the prevalence as 0.5 and 5% margin of error at the 95% confidence interval using the following formula:

(1) $$\begin{array}{c} n=\frac{z_{\alpha /2}^{2}p\left(1-p\right)}{\omega^{2}}, \end{array}$$

where *n* = sample size, *z*=1.96, *p*=0.5, *w*=0.05, CI = 95%, and *α*=5%.

(2) $$\begin{array}{c} n=\frac{{\left(1.96\right)}^{2}\times 0.5\left(1-0.5\right)}{{\left(0.05\right)}^{2}}=384, \end{array}$$

where *n*_f_=*n*/(1+*n*/*N*) in which *N*=601 (estimated target population in the study period).

So

(3) $$\begin{array}{c} n_{\text{f}}=\frac{384}{\left(1+384/601\right)}=234. \end{array}$$

We added 10% of *n*_f_ for the nonresponse rate (i.e., 234 + 23 = 257).

Therefore, a total sample size of 257 elective surgical patients were planned to participate in this study.

### 4.6. Sampling Technique

Since there is no evidence that supports the difference on incidence and predictive values of preoperative tests for difficult laryngoscopy and intubation in seasons or months, we used a consecutive sampling technique. So all eligible patients in the study period were included in this study.

### 4.7. Study Variables

#### 4.7.1. Dependent Variables

The following are the dependent variables:

- Difficult laryngoscopy
- Difficult intubation

#### 4.7.2. Independent Variables

Independent variables are as follows:

1. Sociodemographic variables:
  - Age
  - Sex
  - ASA physical status
2. Airway-related variables (tests):
  - Interincisor distance
  - Mallampati class
  - Thyromental distance
  - Mandibular protrusion
  - Sternomental distance
  - Laryngoscopic grade
3. Anesthesia-related variables:
  - Premedication used and its dose
  - Drug used for induction and its dose
  - Muscle relaxant used and its dose
  - Qualification of the anesthetist
  - Type and size of the laryngoscope
  - External laryngeal pressure

### 4.8. Data Collection Process and Technique

Half-day training was given for qualified anesthetists, who were involved in the data collection process. Structured questionnaire was prepared and tested on 5% of the sample size at the actual study area. Informed consent was taken from each patient orally before data collection, and then patients scheduled for elective surgery under GA requiring endotrachial intubation was assessed at the waiting room by the trained data collectors immediately before their entry to the operation room and recorded on the structured questionnaire. Then, each patient was observed for difficult laryngoscopy and intubation in the operation room. The Cormack and Lehane's laryngoscopic grade of the patient was determined by the anesthetist who performed the laryngoscopy. Observational data collection technique was used in this study.

### 4.9. Data Quality Assurance

Training has given for the data collectors, and the questioner was tested on 5% of the calculated sample size. During the data collection process, there was close supervision of data collectors, and collected data were checked every day for its completeness, clarity, and consistency by the principal investigator.

### 4.10. Data Analysis

Data with complete information were entered to Epi info version 3.4. and then exported to SPSS version 20 for analysis. The descriptive statistics, binary logistic regression, and ROC curve were performed using SPSS. The association b/n independent factors and the outcome variables were determined by chi-square, *p* value, and odds ratio. *Thepvalue of less than 0.05 was considered as statistically significant*. A binary logistic regression and multivariate analysis were computed to assess the independent predictive factors and strength of association between the outcome and explanatory variables. The validity of parameters (screening tests) such as sensitivity, specificity, odds ratio, and 95% confidence intervals was performed using crosstabs on SPSS, whereas positive predictive values and negative predictive values were calculated manually from the descriptive statistics. The effect of combining different parameters on the validity was also analyzed manually.

### 4.11. Dissemination and Utilization of Results

The result will be presented on workshops, Ethiopian Association of Anesthetists Annual Conference, and Scientific Conferences. It will also be discussed with the health managers of Tikur Anbessa specialized hospital. Recommendations will be given to the anesthesia professionals and concerned health practitioners depending on the result. Efforts will be made for publication of the result.

### 4.12. Ethical Considerations

Before conducting the study, ethical clearance was obtained from the Department of Anesthesia ethical review committee. Then, a formal letter detailing the objective of the study was given to the hospital administrators. Then, after getting permission from the hospital managers, data collectors obtained informed consent orally from each patient to collect data. During the data collection process, norms, values, and morals of patients were respected by the data collectors.

### 4.13. Operational Definitions

  Difficult laryngoscopy: Cormack and Lehane grade III (epiglottis only) or grade IV view (soft palate only)
  Difficult intubation: if a trained anesthetist using direct laryngoscopy takes more than 3 attempts or more than 10 minutes to complete tracheal intubation [16].
  General anesthesia: medically induced loss of consciousness and loss of protective reflexes resulting from administration of one or more general anesthetic agents
  American Society of Anesthesiologists (ASA) physical status: it is a method of categorizing patients' physical state developed by the ASA taskforce which classifies patients according to their physical status (systemic wellbeing). It is classified into six classes

#### 4.13.1. Class Definition

  ASA 1: normal healthy patient
  ASA 2: patients with mild systemic disease (no functional limitations)
  ASA 3: patients with severe systemic disease (some functional limitations)
  ASA 4: patients with severe systemic disease that is a constant threat to life (functionality incapacitated)
  ASA 5: moribund patient who is not expected to survive without the operation
  ASA 6: brain-dead patient whose organs are being removed for donor purposes
  E: if the procedure is an emergency, the physical status is followed by “E” (for example, “2E”)
  Interincisor distance (IID): it is the distance between the upper and lower incisors. A value of less than 3 patient's fingers or less than 4 cm predicts difficult airway
  Mandibular protrusion (MP): the lower incisors can be brought in front of the upper incisors, and inability to bring the lower incisors to the upper or mandibular protrusion class B and C suggests difficulty
  Mallampati class (MMC): the Mallampati classification correlates tongue size to pharyngeal size. This test is performed with the patient in the sitting position, head in a neutral position, the mouth wide open, and the tongue protruding to its maximum. Classification is assigned according to the extent the base of the tongue is able to mask the visibility of pharyngeal structures into four classes I–IV:
  Class I: visualization of the soft palate, fauces, uvula, and anterior and the posterior pillars.
  Class II: visualization of the soft palate, fauces, and uvula.
  Class III: visualization of soft palate and base of uvula.
  In Samsoon and Young's modification (1987) of the Mallampati classification, class IV was added.
  Class IV: only hard palate is visible. Soft palate is not visible at all. Mallampati class III and IV suggests difficult laryngoscopy. Class 3 or 4 suggests a significant chance that the patient will be difficult to intubate.
  Thyromental distance (TMD): it is defined as the distance from the mentum to the thyroid notch, while the patient's neck is fully extended. A value of less than 6 cm predicts difficult laryngoscopy or intubation
  Sternomental distance (SMD): it is the distance from the suprasternal notch to the mentum and measured with the head fully extended on the neck with the mouth closed. A value of less than 12 cm is found to predict a difficult intubation.
  Cormack and Lehane laryngoscopic grade: difficulty in intubation can be classified according to the view obtained during direct laryngoscopy into 4 grades. These 4 grades of laryngoscopic views were defined by Cormack and Lehane (1984):
  Grade I—visualization of entire laryngeal aperture.
  Grade II—visualization of only posterior commissure of the laryngeal aperture.
  Grade III—visualization of only epiglottis.
  Grade IV—visualization of just the soft palate.
  N.B: grade III and IV are considered as difficult laryngoscopy and will lead to difficult intubation.

## 5. Results

### 5.1. Sociodemographic Data and Magnitude of Difficult Laryngoscopy and Intubation in the Study Area

A total of 242 patients (129 male and 113 female) were participated in our study. 15/257 (5.8%) patients were excluded from analysis due to incomplete information.

Patients with ASA physical status I to III and age ≥18 years old were included in the study. The mean age of our study population was 38.45 ± 14.869.

**Figure 2:**
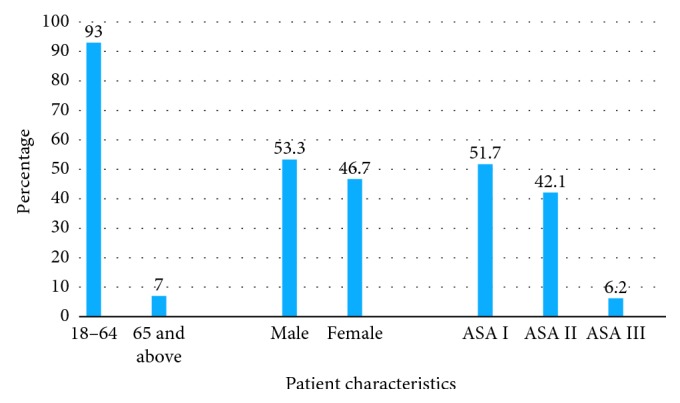
Sociodemographic characteristics of the study population from February 1 to March 30, 2016.

In this study, we found the magnitude of difficult laryngoscopy and intubation as 33/242 (13.6%) and 12/242 (5%), respectively. In this study, difficult intubation was defined as number of attempts ≥4 times based on ASA definition. Only two patients took more than 10 minutes to complete endotracheal intubation. There were no cases with failed intubation (Figure 2 and Table 1).

From Table 1, we can see that the magnitude of difficult laryngoscopy and intubation is higher in the age group of ≥65 years. We found an association between age group of patients ≥65 years and difficult laryngoscopy (*p* value = 0.021 and OR = 3.6 at 95% CI) but not with difficult intubation. ASA class III patients showed an association with difficult intubation (*p* value = 0.033 and OR = 2.7 at 95% CI). Sex of the patients has not showed significant association with both difficult laryngoscopy and intubation (Table 2).

### 5.2. Predictive Values of Preoperative Tests for Difficult Laryngoscopy and Intubation

From Table 2, we can see that 60.7% of patients with MMC III and IV were encountered difficult laryngoscopy of which 25% were also difficult for intubation. From the total of 242 patients, 33/242 (13.6%) were found to be CL III and IV of which 11 (33.3%) of them were also difficult for intubation.

A binary logistic regression showed that Mallampati class and thyromental distance as independent predictors for difficult laryngoscopy with the *p* value = 0.000 and 0.017, respectively, at 92% Hosmer and Lemeshow test.

Patients with Mallampati class III and IV have 12.5 times risk to be difficult for laryngoscopy (AOR = 12.5). Similarly, Cormack and Lehane laryngoscopic grade III and IV, Mallampati class III and IV, and sternomental distance less than 12 cm were identified as independent predictors for difficult intubation (*p* value = 0.000, 0.031, and 0.008, respectively) at 82.7% Hosmer and Lemeshow test. The adjusted odds ratio for SMD < 12 cm, CL = III and IV, and MC = III and IV include 48.9, 30.6, and 7.1, respectively, at 95% confidence interval (Table 3).

From Table 3, we can see that the Mallampati class showed greater accuracy (73.1%) followed by mandibular protrusion (65.6%). All tests showed that greater specificity and negative predictive values than sensitivity and positive predictive values, respectively. Based on Table 3, the receiver operating characteristics curve was used to compare the accuracy of preoperative tests for difficult laryngoscopy.

**Figure 3:**
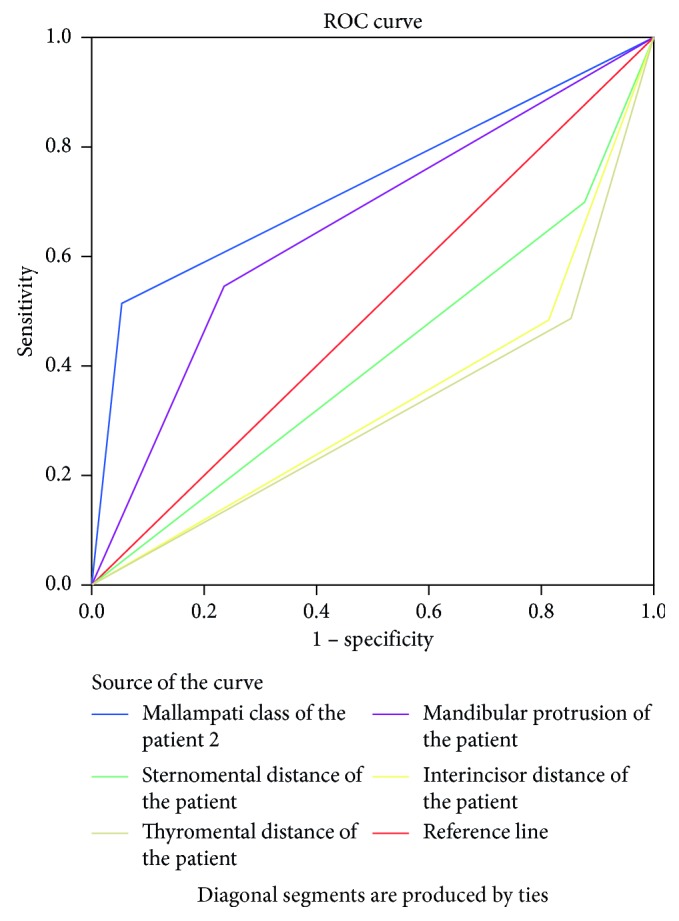
Receiver operating curve for preoperative tests against difficult laryngoscopy in the study population.

The ROC curve showed the Mallampati class and mandibular protrusion above the reference line (0.5) with area under the curve of 0.731 and 0.656, respectively (Figure 3 and Table 4).

**Figure 4:**
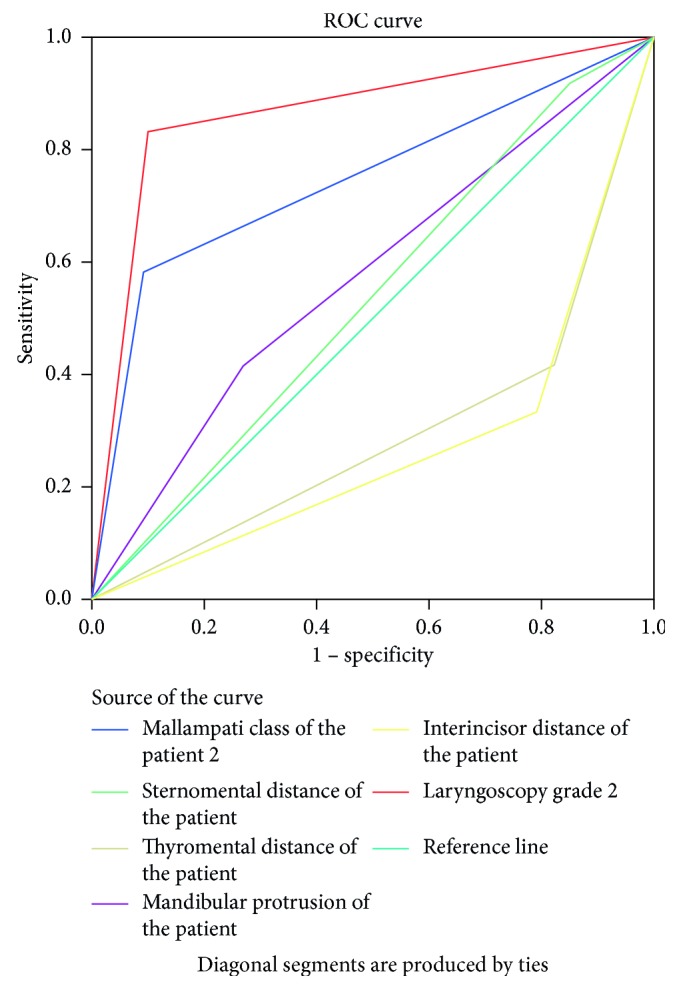
Receiver operating curve for preoperative tests against difficult intubation in the study population.

In Table 4, we can see that higher accuracy to predict difficult intubation in laryngoscopic grade III and IV, Mallampati class III and IV, and mandibular protrusion B and C (86.7%, 74.6%, and 57.4%, respectively). Similar to that of difficult laryngoscopy, preoperative tests for difficult intubation also showed higher specificity and negative predictive values than sensitivity and positive predictive values. Cormack and Lehane laryngoscopic grade III and IV and interincisor distance less than 4 cm have showed greater sensitivity (83.3% and 66.7%, respectively) when compared to other tests. The receiver operating characteristics curve below revealed laryngoscopic grade, Mallampati class, and mandibular protrusion above the reference line (0.5) with the area under the curve of 0.867, 0.746, and 0.574, respectively. The sternomental distance is almost along the reference line with the area under the curve of 0.534 (Figure 4 and Table 5).

### 5.3. Combination of Two Preoperative Tests to Predict Difficult Laryngoscopy and Intubation

From Table 5, we can see that combination of MMC and MP tests has higher sensitivity (78%) for DL followed by combination of MMC and TMD or MMC and IID. Combination of MMC and SMD showed higher specificity (82.9%) but lower sensitivity (66.2%) for DL, whereas MMC + CL has showed the highest sensitivity and specificity for difficult intubation (93% and 81.8%, respectively). MMC + IID also showed higher sensitivity (86.1%) for difficult intubation followed by MMC + SMD.

## 6. Discussion

In this study, we found the magnitude of difficult laryngoscopy and intubation as 13.6% and 5%, respectively. Literature review from 1990 to 1996 reported as difficult direct laryngoscopy occurs in 1.5–8.5% of general anesthetics, and difficult intubation occurs with a similar incidence [3, 8]. Smita et al. [4] found 9.7% and 4.5% difficult laryngoscopy and intubation, respectively. Iohom et al. [12] and Merah et al. [13] reported difficult laryngoscopy as 9% and 3.4%, respectively. The magnitude of difficult laryngoscopy in our study appeared to be higher compared to the available literatures [3, 4, 8, 12, 13]. The probable explanation for this result may be because our study was conducted in teaching hospital, and most of the intubations were performed by undergraduate students (55%). This may lead to incorrect grading of laryngoscopic view due to lack of skill.

Our finding on the difficult intubation was in line with the above studies and with studies by Savva et al. [10], Khan et al. [14], and Yildiz et al. [17] who reported as 4.9%, 5%, and 4.8%, respectively. In contrast to the above findings, Bilgin and Özyurt [18], Keyvan et al. [6], Bhavdip et al. [7], and Tse et al. [9] were reported higher incidence of difficult intubation (8%, 8.24%, 8.1%, and 13.1%, respectively). This may be because of differences in the population characteristics and differences on the definition of DI; for instance, Tse et al. and Shiga et al. were defined DI as laryngoscopic grade III and IV. But, in our study, we defined DI as number of intubation attempts ≥4 times based on ASA definition [1, 2].

In our study, Pearson's correlation showed a positive correlation between age of patients and difficult laryngoscopy (*p* value = 0.021) but not for difficult intubation. In contrast to the difficult laryngoscopy, we found positive correlation between ASA physical status of the patient and difficult intubation with a *p* value of 0.033. So, according to our study, patients with age ≥65 years and ASA III were found to be at higher risk to be difficult laryngoscopy and intubation, respectively. A binary logistic regression identified that Mallampati class and thyromental distance as independent predictors of difficult laryngoscopy with a *p* value of 0.000 and 0.017, respectively, at the 92% Hosmer and Lemeshow test. Patients with Mallampati class III and IV have 12.5 times risk to be difficult laryngoscopy (AOR = 12.5 at 95% CI).

Similarly, we also identified that Cormack and Lehane laryngoscopic grade III and IV, Mallampati class III and IV, and sternomental distance less than 12 cm as independent predictors for difficult intubation (*p* value = 0.000, 0.031, and 0.008, respectively) at the 82.7% Hosmer and Lemeshow test. The adjusted odds ratio for SMD < 12 cm, CL = III and IV, and MC = III and IV include 48.9, 30.6, and 7.1, respectively, at the 95% confidence interval. A multivariate analysis showed that thyromental distance and Mallampati class as an independent predictors for both DL and DI with a *p* value of 0.003 and 0.000 and 0.001 and 0.000, respectively.

This study is comparable with the study conducted by Smita et al. [4]. The mean age of their study was 37.8 ± 13.5, which is comparable with our study (38.45 ± 14.869). They identified MC III and IV and IID ≤ 3.5 cm as independent predictors of difficult laryngoscopy. According to their study, increasing age and male sex have an association with difficult laryngoscopy. Similarly, the study conducted by Yildiz et al. [17] in Turkish patients showed an incidence of difficult intubation as 4.8% and increased with age (*p* < 0.05). They found that the incidence of difficult intubation was significantly higher in patients who had a Mallampati III or IV score, a decreased average thyromental and sternomental distance, decreased mouth opening, or decreased protrusion of the mandible (*p* < 0.05). In our study, sex of the patient does not show association with difficult laryngoscopy and intubation. This finding was in line with the study by Khan et al., and there was no significant difference regarding difficult intubation based on gender, whereas there were significant differences between the older tests and laryngeal view (*p* < 0.05, McNemar test). In contrast to the available literatures, in our study, patients with ASA III were appeared to be at greater risk to develop difficult intubation. This may be due to the medical condition of the patient. Savva et al. [10] conducted a prospective study to predict difficult tracheal intubation on 350 patients, and they found difficult tracheal intubation in 17/350 (4.9%) patients, which was comparable to our finding. According to Srinivasa et al. [19], the *p* value was found to be significant with Mallampati class III & IV, IID, TMD, & SMD for difficult laryngoscopic intubation which is also inline to our study which was in line to our findings, except the IID.

Moreover, our study was also comparable to the study by Khan et al. [14] which was conducted prospectively and showed the prevalence of difficult intubation as 5% (*n*=19). Class III upper lip bite test (MP in our case), IID < 4.5 cm, TMD < 6.5 cm, and SMD < 13 cm were defined as predictors of difficult intubation. The only difference between our study and Khan et al. was the cutoff points for IID, TMD, and SMD. In our study, we used lower cutoff points than the study by Khan et al. (<4 cm, <6 cm, and <12 cm, respectively). The reason why we prefer the lower cutoff points were to decrease false-positive rates. Different authors used different cutoff points for the preoperative airway tests. This may be because of the difference in the populations' physical appearance and ethnic group [4]. So, this suggests that we should have to develop our own cutoff points for the airway parameters in our country to predict difficult laryngoscopy and intubation more precisely.

Concerning the predictive values of the independent variables or tests, our study showed poor sensitivities and positive predictive values but good specificity and negative predictive values for both difficult laryngoscopy and intubation. The sensitivity, specificity, and positive predictive values of IID, MMC, MP, TMD, and SMD for difficult laryngoscopy in our study showed that (51.5%, 81.3%, and 30.35%), (51.5%, 94.7%, and 60.7%), (54.5%, 76.6%, and 26.8%), (51.5%, 85.2%, and 35.4%), and (30.3%, 87.6% & 27.7%), respectively. Similarly, the sensitivity, specificity, and positive predictive values of IID, MMC, MP, TMD, SMD, and CL for difficult intubation were (66.7%, 79.1%, and 14.3%), (58.3%, 90.9%, and 25%), (41.7%, 73%, and 7%), (58.3%, 82.2%, and 14.6%), (8.3%, 84.8%, and 27.7%), and (83.3%, 90%, and 30.3%), respectively, which was comparable to Shiga et al. and most of the available literatures [12–14,20]. Except the SMD, all tests were statistically significant for DL (*p* value <0.05) at 95% confidence interval, whereas CL has showed greater sensitivity, specificity, and positive predictive values for DI followed by IID. MP and SMD showed insignificant *p* value (*p* > 0.05) for difficult intubation even though they have good specificity and negative predictive values. There were limited data that showed the predictive values of preoperative tests for difficult laryngoscopy and difficult intubation separately.

This study is comparable with the study conducted by Iohom et al. [12], where they found poor sensitivity and positive predictive values for MC, TMD, IID, and SMD. Similarly, Merah et al. [13] found the sensitivity, specificity, and the positive predictive values for the five airway predictors as follows: MMT (61.5%, 98.4%, 57.1%), TMD (15.4%, 98.1%, 22.2%), SMD (0%, 100%, 0%), HLM (30.8%, 76.0%, 4.3%), and IIG (30.8%, 97.3%, 28.6%), which shows poor sensitivity and positive predictive values but better specificity. According to Shiga et al. [20], screening tests included were Mallampati classification, thyromental distance, sternomental distance, mouth opening, and Wilson risk score. Each test yielded poor to moderate sensitivity (20–62%) and moderate to fair specificity (82–97%).

In contrast to the above findings, Srinivasa et al. [19], Bhavdip et al. [7], and Savva [10] showed greater sensitivity, specificity, and positive predictive values for most of the above tests. This may be due to differences in patients' physical appearance, sample size, and cutoff values for the screening tests.

In our study, Mallampati class showed better accuracy (73.1%) followed by mandibular protrusion (65.6%) for difficult laryngoscopy, whereas Mallampati class III and IV and Cormack and Lehane laryngoscopic grade III and IV showed good accuracy for difficult intubation (86.7% and 74.6%), respectively. The available literatures did not show the predictive value of difficult laryngoscopy for difficult intubation.

Currently available screening tests for difficult intubation have only poor to moderate discriminative power when used alone. So, combinations of tests add some incremental diagnostic value in comparison to the value of each test alone [21]. Literatures have also recommended that the use of combined preoperative measurement parameters in predicting difficult intubation [4, 12, 13, 22, 23].

In a study with a large sample size, researchers noted that the combination of MMT (MMC) and TMD were good predictors of a difficult laryngoscopy in the Thai population [24, 25]. They used TMD < 6 cm as a parameter as we used in the current study.

Iohom et al. [7] were also performed a study in Ireland and noted that the validity of positive predictive value of MMT (MMC) increased from 27 to 100% after combining other predictors. The Mallampati test is a worldwide used scoring system for predicting difficult intubation. It has been cited in numerous publications since 1985. It still remains a clinical assessment method for many anesthesiologists despite some controversies on its predictive ability.

In our study, we analyzed the validity of combination of the Mallampati test with other preoperative tests when performed simultaneously to better predict difficult laryngoscopy and intubation. Combination of MMC and MP followed by combination of MMC and TMD/IID showed improved sensitivity for DL (78% and 76.4%), respectively, and Combination of MMC and SMD followed by MMC and TMD showed improved specificity for DL (82.9% and 80.6%), respectively. Combination of MC and CL followed by MMC and IID showed improved sensitivity for difficult intubation (93% and 86.1%, respectively), whereas combination of MMC and CL followed by MMC and SMD showed improved specificity (81.8% and 77%, respectively). In our study, combination of MMC with MP and MMC with IID/TMD appeared to be more sensitive and best combinations for prediction of difficult laryngoscopy and intubation in our study population. Because the combinations of MMC with TMD/IID showed good sensitivity and specificity for both DL and DI, combinations of MMC, TMD, and IID can be more valuable preoperative tests in predicting difficult laryngoscopic intubation. MMC and TMD were also appeared to be independent predictors for both difficult laryngoscopy and intubation in the multivariate analysis in our study.

These findings were in line with the study by Merah et al. [13], who's best combination of predictors were MMT/TMD/IIG with a sensitivity, specificity, and positive predictive value of 84.6%, 94.6%, and 35.5%, respectively. Logistic regression analysis showed that weight, MMT, IIG, and TMD were independent predictors of DVL. They concluded that MMT (i.e., MMC), TMD, and IIG appear to provide the optimal combination in prediction of DVL in the West African population [13]. Bhavdip et al. [7] also suggested that the combination of MMT (MMC) grading and TMD and SMD measurements increased the validity (sensitivity of 100% and specificity of 92.7%) [7]. Iohom et al. [12] suggested that the Mallampati classification, in conjunction with measurement of the thyromental and sternomental distances, may be a useful routine screening test for preoperative prediction of difficult tracheal intubation [12].

Similarly, Shiga et al. [20] suggested that the most useful bedside test for prediction was found to be a combination of the Mallampati classification and thyromental distance (positive likelihood ratio, 9.9; 95% confidence interval, 3.1–31.9).

On the contrary, Khan et al. [14] found the combination of the ULBT (i.e., MP) with SMD provided the highest sensitivity. They concluded that the specificity and accuracy of the ULBT is significantly higher than the other tests and is more accurate in airway assessment. However, the ULBT in conjunction with the other tests could more reliably predict easy laryngoscopy or intubation [14]. Our study does not support this conclusion. This might be again because of the effect of physical characteristics of the study population and cut point values. Differences in the definition of difficult intubation may contribute to the difference in the magnitude of DI, and this intern may play a role for the variation of predictive values of preoperative tests among different studies as we have seen from the above findings.

### 6.1. Limitations of the Study

The limitations of our study were as follows: (1) most of the laryngoscopies and intubations were performed by undergraduate students, and this might have contributed for higher magnitude of difficult laryngoscopy. (2) Airway management may not follow standard guideline, or there may be interpersonal variations in the anesthetic management in terms of their experience, preparation, and availability of equipment for intubation which may also have an impact on the magnitude of difficult laryngoscopy and intubation. (3) Lack of standardized cutoff values for the preoperative airway parameters. Different authors used different cutoff values for preoperative tests which can impose some difficulties in comparing different findings.

## 7. Conclusion

We found magnitude of 13.6% and 5% for difficult laryngoscopy and intubation, respectively, among elective surgical patients with apparently normal airways in Tikur Anbessa Specialized Teaching Hospital. Increasing age and ASA class III showed association with difficult laryngoscopy and intubation, respectively. Binary logistic regression identified that Mallampati class and thyromental distance as an independent predictors for difficult laryngoscopy with a *p* value = 0.000 and 0.017, respectively. Similarly, laryngoscopic grade III and IV, Mallampati class III and IV, and sternomental distance less than 12 cm were identified as an independent predictors for difficult intubation (*p* value = 0.000, 0.031, and 0.008, respectively). A multivariate analysis identified Mallampati class and thyromental distance as an independent predictors for both difficult laryngoscopy and intubation with a *p* value of (0.003 and 0.000) and (0.001 and 0.000), respectively.

The sensitivity and positive predictive values of individual preoperative tests appeared to be poor but showed good specificity and negative predictive values. The ROC curve revealed better accuracy with Mallampati class and mandibular protrusion to predict difficult laryngoscopy with the area under the curve of 0.731 and 0.656, respectively, at 95% confidence interval. Similarly, Mallampati class and laryngoscopic grade showed more accuracy to predict difficult intubation with the area under curve of 0.746 and 0.867, respectively. Combination of modified MC with IID/TMD/CL showed improved sensitivity and specificity and was found to be better to predict difficult laryngoscopy and intubation.

### 7.1. Recommendations

Inspite of various airway assessment tests, no single test was 100% accurate. So it is advisable to use combination of different tests.

We would like to recommend anesthesia professionals to use the combination of MC/TMD/IID as their routine preoperative tests to predict difficult laryngoscopic intubation.

Anesthesia professionals should develop guideline for preoperative airway assessment to decrease incidence of difficult laryngoscopy and intubation. Further multicenter study should be conducted in this particular topic to develop national guideline for preoperative airway assessment.

## Figures and Tables

**Table 1:** Demographic characteristics and distribution of difficult laryngoscopy and intubation among surgical patients who underwent elective surgery in Tikur Anbessa Hospital from February 1 to March 30, 2016.

Patient characteristics	Frequency, *n* (%)	DL, *n* (%)	DI, *n* (%)
Age			
18–64	225 (93)	27 (12)	11 (4.8)
≥65	17 (7)	6 (35.3)	1 (5.8)

Sex			
Male	129 (53.3)	18 (13.9)	5 (3.8)
Female	113 (46.7)	15 (13.3)	7 (6.2)

ASA status			
I	125 (51.6)	12 (9.6)	3 (2.4)
II	102 (42.1)	20 (19.6)	7 (6.8)
II	15 (6.2)	1 (6.6)	2 (13.3)

**Table 2:** Preoperative airway parameters and their distribution with difficult laryngoscopy and intubation among surgical patients in Tikur Anbessa Hospital from February 1 to March 30, 2016.

Parameter	Frequency, *n* (%)	DL, *n* (%)	DI, *n* (%)
IID			
<4 cm	56 (23.1)	17 (30.3)	8 (14.3)
≥4 cm	186 (76.9)	16 (8.6)	4 (2.1)
MP			
A	175 (72.3)	15 (8.5)	7 (4)
B and C	67 (27.7)	18 (26.8)	5 (7.4)
MMC			
I and II	214 (88.4)	16 (7.5)	5 (2.3)
III and IV	28 (11.6)	17 (60.7)	7 (25)
TMD			
<6 cm	48 (19.8)	17 (35.4)	7 (14.5)
≥6 cm	194 (80.2)	16 (8.2)	5 (2.5)
SMD			
<12 cm	36 (14.9)	10 (27.7)	1 (2.7)
≥12 cm	206 (85.1)	23 (11.1)	11 (5.3)
CL			
I and II	209 (86.4)	0	1 (0.47)
III and IV	33 (13.6)	33 (13.6)	11 (33.3)

**Table 3:** Sensitivity, specificity, positive predictive values, and negative predictive values for preoperative parameters against difficult laryngoscopy among surgical patients in Tikur Anbessa Hospital from February 1 to March 30, 2016.

Parameter	Sn (%)	Sp (%)	PPV (%)	NPV (%)	Area	*p* value	95% CI	Accuracy (%)
IID	51.5	81.3	30.35	91.4	0.336	0.002	0.228–0.444	33.6
MP	54.5	76.6	26.8	91.4	0.656	0.004	0.549–0.762	65.6
MC	51.5	94.7	60.7	92.5	0.731	0.000	0.621–0.841	73.1
TMD	51.5	85.2	35.4	91.7	0.317	0.001	0.208–0.425	31.7
SMD	30.3	87.6	27.7	88.8	0.411	0.099	0.298–0.523	41.1

Sn = sensitivity; Sp = specificity; PPV = positive predictive value; NPV = negative predictive value; CI = confidence interval; *p* < 0.05.

**Table 4:** Sensitivity, specificity, positive predictive values, and negative predictive values for airway parameters against difficult intubation among surgical patients in Tikur Anbessa Hospital from February 1 to March 30, 2016.

Parameters	Sn (%)	Sp (%)	PPV (%)	NPV (%)	Area	*p* value	95% CI	Accuracy (%)
IID	66.7	79.1	14.3	97.3	0.271	0.008	0.112–0.430	27.1
MP	41.7	73	7.4	96	0.574	0.391	0.401–0.746	57.4
MMC	58.3	90.9	25	97.6	0.746	0.004	0.574–0.918	74.6
TMD	58.3	82.2	14.6	97.4	0.297	0.018	0.129–0.466	29.7
SMD	8.3	84.8	27.7	94.6	0.534	0.688	0.375–0.694	53.4
CL	83.3	90	30.3	99	0.867	0.000	0.742–0.991	86.7

Sn = sensitivity; Sp = specificity; PPV = positive predictive value; NPV = negative predictive value; CI = confidence interval; *p* < 0.05.

**Table 5:** Sensitivity and specificity of combined preoperative tests for difficult laryngoscopy and intubation among surgical patients in Tikur Anbessa Hospital from February 1 to March 30, 2016.

Parameters	Sn (%)		Sp (%)	
	DL	DI	DL	DI
MMC + MP	78	75.6	72.5	66.3
MMC + SMD	66.2	61.7	82.9	77
MMC + TMD	76.4	82.6	80.6	74.7
MMC + IID	76.4	86.1	76.9	71.9
MMC + CL	—	93	—	81.8

Sn = sensitivity; Sp = specificity.

## Data Availability

The data used to support the findings of this study are available from the corresponding author upon request.

## Abbreviations

ASA: American Society of Anesthesiologists
AUC: Area under the curve
DA: Difficult airway
DAS: Difficult airway society
DI: Difficult intubation
DL: Difficult laryngoscopy
ETT: Endotracheal tube
GA: General anesthesia
IID: Interincisor distance
IIG: Interincisor gap
LEMON: Look externally, evaluate 3-3-2, Mallampati class, obstruction, and neck mobility
MMC: Modified Mallampati class
MMT: Modified Mallampati test
MOH: Ministry of Health
MP: Mandibular protrusion
ROC: Receiver operating characteristics
SMD: Sternomental distance
TASH: Tikur Anbessa Specialized Hospital
TMD: Thyromental distance
ULBT: Upper lip bite test.

## Supplementary Materials

Supplementary Materials This supplementary material consists of the title of the research, information provided during the informed consent for data collection which was both in English and Amharic languages, questionnaire (which assesses patient identification, airway parameters, and anesthesia-related data), receiver operator characteristics (ROC) curve and its classifications (which is used to assess the accuracy of airway tests in predicting difficult laryngoscopy and intubation), and declaration of the researchers. The material may help other researchers conducting similar studies as a reference material or as a start point and could be modified as necessary.
