# Supplementary material for: Predictive Values of Preoperative Tests for Difficult Laryngoscopy and Intubation in Adult Patients at Tikur Anbessa Specialized Hospital
Source: Anesthesiol Res Pract. 2019 Apr 1;2019:1790413. doi: 10.1155/2019/1790413 (PMC6466924; doi:10.1155/2019/1790413)
Supplement: Supplementary Materials — This supplementary material consists of the title of the research, information provided during the informed consent for data collection which was both in English and Amharic languages, questionnaire (which assesses patient identification, airway parameters, and anesthesia-related data), receiver operator characteristics (ROC) curve and its classifications (which is used to assess the accuracy of airway tests in predicting difficult laryngoscopy and intubation), and declaration of the researchers. The material may help other researchers conducting similar studies as a reference material or as a start point and could be modified as necessary. [file 1790413.f1.pdf]

Annexes

I. <u>Consent Form</u>

Addis Ababa University, College of Health Sciences Department of Anesthesia

Magnitude and Predictive Value of Preoperative Tests for Difficult Laryngoscopy and Intubation among Surgical Patients who underwent elective Surgery under General Anesthesia in Tikur Anbessa Specialized Hospital from Feb.1- March 30, 2016.

Introduction

My Name is………….. , I work for ……………….

This questionnaire is to assess the magnitude and predictive value of preoperative tests for difficult laryngoscopy and intubation among surgical patients of Tikur Anbessa Specialized Hospital. The main concern of this study was to fill the information gap on the magnitude and predictive value of preoperative tests for estimating difficult laryngoscopy and intubation which can contribute to improve perioperative patient management and decrease anesthesia related morbidity and mortality. So you are kindly requested to participate on this study and provide appropriate response to questions. We obtained your name from the list of appointed surgical patients. Your participation is voluntary. Only anonymous data will be analyzed and we strictly keep confidentiality of participants. Participating or not participating on this study will not bring any harm or benefit to you. This interview will take a maximum of 15 minutes. Therefore we kindly request you to respond for the following questions based on your willingness. If you feel or face any problem regarding your participation, you can contact the principal investigators by 0913724684.

Can I proceed? Yes --------- No -----------

Thank you for your participation!

<u>የአማርኛጥያቄቅፅ</u>

ሰላም፣ እኔ ____________________ እባላለሁ። የምሰራዉነዉ።

በቀዶጥገናጊዜየሰመመንህክምናሲጀመርየኣየርቧንቧቱቦከማስገባትጋርተያይዞየሚፈጠርየአየር

ቧንቧችግርላይጥናትበማድረግላይእንገኛለን።

እኔምየቡድኑአንድአካልነኝ።የተመደበዉሀኪምየአየርባንባቱቦዉንበተገቢዉየአካልክፍልሲያስገባና

የዚህጥናት አላማ የችግሩን ስፋትና አጋላጭነገሮችን ለማወቅ ነዉ።

ይህጥናትየበሽተኛዉንደህንነትናየችግሮቹንአስከፊነትለመቀነስናአጋላጭምክንያቶቹንእንዲሁምየችግሩንጥ

ልቀትናአሳሳቢንትበሆስፒታሉለማሳወቅነዉ።

በዚህጥናትሂደትውስጥየሚሰበሰበውማንኛውምአይነትመረጃሙሉበሙሉበምስጢርየሚጠበቅመሆኑንል

ናረጋግጥልዎእንወዳለን።የማንኛውምተሳታፊስምከግምትውስጥየማይገባናየሚሰጡትምማንኛውንምመ

ረጃለማንምተላልፎእንደማይሰጥብሎምየዚህጥናትውጤትእርሶንበስምእንደማይለይዎትእናረጋግጣለን።

መጠይቁበፈቃደኝነትላይየተመሰረተሲሆንእርሶበዚህጥናትላይየመሳተፍሙሉመብትአለዎት።ይህምብቻሳ

ይሆንከጀመሩበኋላምበማንኛውምጊዜማቋረጥእንዲሁምሙሉበሙሉያለመሳተፍመብትአለዎት።

መቀጠል እችላለሁ;

1. አዎ ይቻላል

2. አይቻልም

Informed consent Certified by

Name________________________ signature_____

Date of data collection ____________

Questionnaire identification number__________________

II. <u>Questionnaire</u>

Part I: Identification

Code No ………… Age:……. yrs Sex: male ☐ female ☐

ASA status: I II III IV V

Part II: Airway characteristics

1. Interincisor distance: A. less than 4cm/ 3 patients' fingers ☐ B. greater than or equal to 4cm/ 3 patient's fingers ☐
2. Mallampatti class: I II III IV
3. Mandibular protrusion: A B C
4. Thyromental distance: A. less than 6cm ☐ B. greater than or equal to 6cm ☐
5. Sternomental distance: A. less than 12cm ☐ B. greater than or equal to12cm ☐
6. Cormack & Lehane laryngoscopic grade: I II III IV
7. External laryngeal pressure: A. applied ☐ B. not applied ☐
8. Number of attempts for intubation: I II III IV
9. Time taken to intubate the patient: A. less than 10 minutes B. greater than or equal to 10 minutes
10. Intubation : A. Failed ☐ Successful ☐
11. Qualification of the anesthetist who performed the intubation: A. under graduate student B. Msc in Anesthesia student C. Bsc Anesthetist D. Msc Anesthetist E. anesthesiology resident F. Anesthesiologist

Part III: Anesthetic Technique

1. Premedication used ……… Not used…….
2. Drug used for induction and its dose …………….
3. Drug used for muscle relaxation and its dose …………
4. Type and size of laryngoscopy blade ………………
5. External laryngeal pressure: A. applied B. not applied

III. ROC Curve

The ROC curve is a graph of sensitivity (y-axis) vs. 1 – specificity (x-axis). The curve is created by plotting the true positive rate against the false positive rate at various threshold settings. The true-positive rate is also known as sensitivity. The false-positive rate is also known as the fall out and can be calculated as 1 – specificity.

The accuracy of the test depends on how well the test separates the group being tested into those with and without the condition of interest.

The diagonal line y = x represents the strategy of randomly guessing a class. This reference line is a random classifier which produce a ROC point that ‘‘slides’’ back and forth on the diagonal based on the frequency with which it guesses the positive class. It is also known as line of equality or random chance line.

Accuracy is measured by the area under the ROC curve (AUC). ROC curve areas are typically between 0.5 and 1.0. An area of 1 represents a perfect test; an area of 0.5 represents a worthless test.

Simple guide for classification ROC curve areas:

- .90-1 = excellent (A)
- .80-.90 = good (B)
- .70-.80 = fair (C)
- .60-.70 = poor (D)
- .50-.60 = fail (F)

IV. Declaration

I, the undersigned, declare that this thesis is my original work in partial fulfillment of the requirements for the degree of MSc in Anesthesia. I understand that plagiarism will not be tolerated and all cited materials have been appropriately referenced.

Name: ____________________

Signature: ____________________

To be submitted to the Department of Anesthesia, Addis Ababa University

Date of Submission: ________________________________

This thesis work has been submitted for examination with my/our approval as Advisor on the MSc in Anesthesia course.

Name	Signature
1.	
2.	
